# Immunomics in Pediatric Rheumatic Diseases

**DOI:** 10.3389/fmed.2019.00111

**Published:** 2019-05-29

**Authors:** Shi Huan Tay, Katherine Nay Yaung, Jing Yao Leong, Joo Guan Yeo, Thaschawee Arkachaisri, Salvatore Albani

**Affiliations:** ^1^Duke-NUS Medical School, Singapore, Singapore; ^2^Translational Immunology Institute, SingHealth Duke-NUS Academic Medical Centre, Singapore, Singapore; ^3^Rheumatology and Immunology Service, Department of Pediatric Subspecialties, KK Women's and Children's Hospital, Singapore, Singapore

**Keywords:** immunomics, rheumatology, genomics, transcriptomics, proteomics, cytomics, epigenomics

## Abstract

The inherent complexity in the immune landscape of pediatric rheumatic disease necessitates a holistic system approach. Uncertainty in the mechanistic workings and etiological driving forces presents difficulty in personalized treatments. The development and progression of immunomics are well suited to deal with this complexity. Immunomics encompasses a spectrum of biological processes that entail genomics, transcriptomics, epigenomics, proteomics, and cytomics. In this review, we will discuss how various high dimensional technologies in immunomics have helped to grow a wealth of data that provide salient clues and biological insights into the pathogenesis of autoimmunity. Interfaced with critical unresolved clinical questions and unmet medical needs, these platforms have helped to identify candidate immune targets, refine patient stratification, and understand treatment response or resistance. Yet the unprecedented growth in data has presented both opportunities and challenges. Researchers are now facing huge heterogeneous data sets from different origins that need to be integrated and exploited for further data mining. We believe that the utilization and integration of these platforms will help unravel the complexities and expedite both discovery and validation of clinical targets.

## Introduction

Unraveling the etiology of pediatric rheumatic diseases exposes the complex heterogeneity inherent within the networks of immune pathophysiology. This mechanistic complexity underscores the challenge and uncertainty in precise disease characterization or sub-stratification. One illustrative example is the International League of Associations for Rheumatology (ILAR) classification of juvenile idiopathic arthritis (JIA) which serves to discriminate seven categories through a combination of clinical presentations, family history, serum, and genetic markers (1). Yet recent advances have shown that the disease mechanisms in JIA patients may be far more diverse (2). The clinical inability to deal with disease heterogeneity manifests as difficulties in confidently predicting responses and matching patients to current available treatment modalities (3). Furthermore, the development of future better-fit therapeutics will require dissecting the plethora of available immunological data through the lens of individual patient-specific clinical information. Cross comparisons of immunological data between different diseases could reveal underlying similarity in immune architecture that would facilitate opportunistic repurposing of existing drugs that have passed phase 1–3 clinical trials (4) and ultimately reduce drug development costs. These challenges and clinical unmet needs necessitate a different approach.

**Figure 1:**
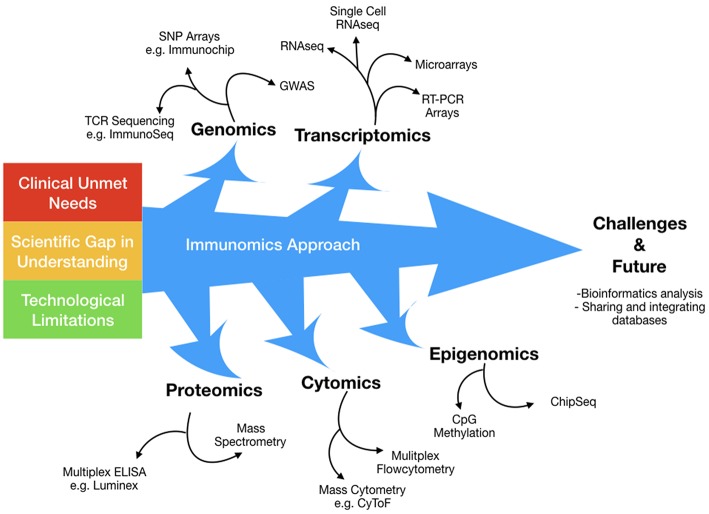
The immunomics approach has evolved over time to surmount technological limitations in a broad attempt to answer several clinical and scientific unmet needs. Immunomics can be conceptualized as the application of high dimensional technologies that aim to harvest information across a spectrum of biological processes, encompassing; genomics, transcriptomics, epigenomics, proteomics and cytomics. This holistic approach is well suited in deciphering key mechanistic information from complex immune networks, thus unveiling insights that may well otherwise be hidden.

Immunomics can be understood as the application of high dimensional technologies that aim to harvest information across a spectrum of biological processes, encompassing (a) genomics, (b) transcriptomics, (c) epigenomics, (d) proteomics, and (e) cytomics (Figure 1). This holistic approach is well-suited to distilling key mechanistic information from complex immune networks, thus providing insights that may well otherwise be hidden. For instance, with greater dimensional resolution, we can now decipher key subtle mechanistic differences away from background stochastic immune features, and increasingly obtain better understanding of intra- and inter-individual pathological diversity.

In this review, we will discuss how conventional high dimensional immunomics platforms, and recent emerging technologies in single cell immune profiling (mass cytometry and single cell transcriptomics) have been deployed in pediatric rheumatic diseases. The resultant explosion of biological information entails corresponding challenges in bioinformatics analysis and public data sharing platforms, and how these issues are addressed will be examined. We believe the incorporation and integration of immunomics platforms in the research community will serve to illuminate and expedite both discovery and clinical validations.

## Genomics

Genetic susceptibility is a cardinal aspect of pediatric rheumatic diseases, and comprehension of how individual genetic variants influence pathogenesis will subsequently guide prognostication and disease management. However, the challenge with pediatric rheumatic diseases is underpinned by its heterogeneity in disease susceptibility, clinical presentations as well as treatment outcomes. Genomics is well-placed to address this quandary by (a) identifying candidate genetic susceptibility loci through an unsupervised genome wide interrogation and (b) by streamlining disease classification into more homogenous subtypes, so that disease pathways may be elucidated and therapeutic selection more personalized. Another facet of immunological heterogeneity pertains to the inter-individual differences in immune repertoire present in T cell receptors (TCR). Advancements in TCR repertoire sequencing will add another dimension in the understanding of why certain individuals develop autoimmune diseases and maintain disease persistence. Major insights have since been gleaned from genetic studies across multiple pediatric rheumatic diseases, thereby augmenting our understanding of these diseases.

### Genome-Wide Association Studies

Genome-wide association studies (GWAS) are hypothesis-free studies in which a dense array of genetic markers, achieving significant representation in the genomic sequence, are instructive for a trait of interest (5). A typical genetic marker is the single nucleotide polymorphism (SNP), which is a variation in a single nucleotide occurring at a specific position along the genome, and some SNPs will be co-inherited with the trait of interest due to proximity along a contiguous stretch of genomic sequence. By detecting these associations between specific SNPs and disease on a population scale and deeming them robust if differences in allelic frequency between cases and controls exceed a statistical genome-wide significance threshold, susceptibility effects can hence be mapped. Given that pediatric rheumatic diseases are genetically complex with multiple genes of low effect sizes as well as gene-gene and gene-environment interactions, GWAS represent a major step forward from prior candidate gene studies and low-powered family linkage studies (Table 1).

**Table 1:** Summary of recent immunomics applications and their impact on our understanding of pediatric rheumatic disease (I).

**Immunomics techniques**	**Clinical application and discovery**	**References**
**Genomics**		
Genome Wide Association Studies (GWAS) with/without Immunochip	JIA susceptibility	
	Multiple HLA loci	(6)
	*VTCN1, STAT4, TRAF1/C5, PTPN22, PTPN2, CD8, and JMJDC1*	(6–10)
	JIA classification	
	HLA-DRB1^*^11, HLA-DRB1^*^04 (systemic JIA), *PTPN22, ATP8B2_IL6R, STAT4, IL2_IL21, ERAP2_LNPEP, HLA, IL2RA, COG6, PTPN2, PRR9_LOR and ILDR1_CD86* (oligoarthritis and RF-negative polyarthritis)	(11–14)
	*VTCN1* (uveitis)	(15)
	JIA with methotrexate response	
	TGF-beta signaling pathway	(16)
	JDM susceptibility	
	HLA DRB1^*^03:01 *PTPN22*	(17, 18)
	Childhood onset SLE susceptibility	
	*XKR6* and *GLT1D1*	(19)
	Kawasaki disease	
	*NEBL* and *TUBA3C* (increased risk of coronary artery lesions)	(20)
	Henoch Schönlein Purpura (HSP) susceptibility	
	HLA class II region (including *HLA-DQA1/DQB1, HLA- DRB1*)	(21)
T-cell receptor (TCR) sequencing	JIA disease activity and treatment response markers	
	Circulating pathogenic-like lymphocytes (CPLs) and inflammation-associated Treg (iaTreg) – persistent disease activity and resistance to methotrexate and anti-TNFα therapy	(22, 23)
	TCR repertoire restoration – autoimmune disease remission after HSCT and sJIA remission	(24–26)

*HLA, Human Leukocyte Antigen; JIA, Juvenile Idiopathic Arthritis; sJIA, systemic JIA; JDM, Juvenile Dermatomyositis; SLE, Systemic Lupus Erythematosus; TGF, Transforming growth factor; Treg, regulatory T cell; HSCT, Hematopoietic Stem Cell Transplantation*.

An early success of GWAS in pediatric rheumatic diseases was the discovery of *VTCN1*, implicated in immune attenuation through B/T lymphocytes, as a novel JIA susceptibility locus in a 2009 study involving 279 JIA cases in the discovery cohort and 321 JIA cases in the validation cohort (7). Several contemporary JIA GWAS studies also built upon the findings of the Wellcome Trust Case Control Consortium Study to add both human leukocyte antigen (HLA) and non-HLA loci to the list, for which the latter included genes involved in T cell regulation and signaling; *STAT4, TRAF1/C5, PTPN22, PTPN2, CD80*, and *JMJDC1* (6, 8–10). This experience is mirrored by the Myositis Genetics Consortium (MYOGEN), through an international collaborative effort that revealed the presence of HLA DRB1^*^03:01 as a disease susceptibility locus for juvenile dermatomyositis (JDM) (17). Since then, the advent of large consortia with their corresponding larger sample sizes, meta-analyses tapping on global databases as well as improvements in GWAS technology have further enhanced our knowledge on disease pathways, classification, and management.

Large-scale meta-analyses, which are statistical studies interrogating the combined results from multiple independent studies, have permitted analysis at an increased power and hence detection of signals that would have otherwise be missed due to their small effect sizes in underpowered single GWAS. This has been of great use in JIA, whereby two studies identified novel susceptibility loci for different subtypes of the disease: HLA-DRB1^*^11 was uncovered as a strong systemic JIA (sJIA) risk factor following a meta-analysis of 9 independent case-control populations consisting 982 patients and 431 healthy children (11), while 9 new oligoarticular and rheumatoid factor (RF)-negative polyarticular JIA loci including *PRR9_LOR* and *ILDR1_CD86* were identified from three cohorts comprising of 2,751 patients and 15,886 controls (13).

Given that associations identified following GWAS and meta-analysis tend to implicate several genetic variants in disease susceptibility, it is imperative to determine the loci with the strongest evidence for candidate causal association. The realization that there is significant overlap in genetic susceptibilities across different autoimmune diseases led to the development of the Immunochip, which is a dedicated SNP array created for fine-mapping 186 autoimmunity loci established from prior GWAS (27). The use of the Immunochip has helped to refine peaks of association identified in previous GWAS and increase sensitivity in discovering new risk loci; in a well-powered study of ~2,000 patients with either oligoarticular or rheumatoid factor (RF)-negative polyarticular JIA, three known JIA risk loci (the HLA region, *PTPN22*, and *PTPN2*) and 14 novel loci reaching genome-wide significance (*p* < 1 × 10^−6^) were uncovered for the first time (14). The same fine-mapping approach, in a separate study involving 14 countries, also supported association of the HLA and *PTPN22* regions for susceptibility to idiopathic inflammatory myopathies (IIM), of which JDM is a major subtype (18). In a systemic lupus erythematosus (SLE) Korean cohort with 781 patients, a hybrid approach with a GWAS array and Immunochip genotyping allowed for the sensitive detection of two SLE risk loci, *XKR6* and *GLT1D1*, which were found to be significantly higher in childhood-onset SLE (19).

In tandem, efforts involving large-scale sequencing projects such as the 1000 Genomes Project have continued to expand the catalog of known population variants, which in turn provide multi-ancestral reference populations from which control genotypes can be readily used from their public databases (28). For instance, a GWAS study conducted on IgA vasculitis, also known as Henoch-Schönlein purpura (HSP) and commonly found in children, with the aid of reference panels from the 1000 Genomes Project, revealed the HLA class II region as the major susceptibility locus (21).

Such advances in the validity of GWAS data and availability of public reference databases have renewed interest in the incorporation of this genetic information in pediatric rheumatic disease classification. The current categorization of JIA is primarily based on clinical signs and symptoms, such as the number of joints affected and the extent of extra-articular manifestations (1), but fails to account for underlying disease pathogenesis and hence helps in neither prognostication nor therapy selection. In particular, sJIA's autoinflammatory inflammatory phenotype clearly distinguishes it from the other 6 subtypes (29). Thus, GWAS comparing genetic variation across different JIA subtypes can help to highlight differences in genetic architecture that can potentially explain the distinct presentation of sJIA. A recent study on 770 patients with sJIA from 9 populations of European ancestry demonstrated a lack of shared genetic susceptibility loci with other JIA subtypes (oligoarthritis and RF-negative polyarthritis), with two susceptibility loci exceeding genome-wide significance: the major histocompatibility complex (MHC) II/III and a region located 20 kb upstream of *LOC284661* (a long intergenic non-coding RNA) loci (30). This is in line with the discovery of HLA-DRB1^*^11 as a strong sJIA risk factor with an overall Odds Ratio (OR) of 2.3, and HLA-DRB1^*^04 with a pooled OR of 1.9 from a meta-analysis of prior candidate gene studies of MHC II in JIA (11, 12). The implications of this result are 2-fold as it not only highlights the genetic dissimilarity between sJIA and other JIA subtypes, but also proposes the concurrent involvement of the adaptive immune system on top of a dysregulated innate immunity in disease pathogenesis. Thus, this paves the way for future mechanistic studies of these susceptibility loci to further elucidate sJIA pathophysiology and subsequently identify better targets for therapy.

GWAS findings have also provided insights into disease outcomes, with both HLA and non-HLA gene polymorphisms emerging as risk factors for certain disease manifestations as well as predictors of therapeutic response. The presence of amino acid serine at position 11 in HLA-DRB1 was shown to confer an increased risk of uveitis (OR 2.60) in female JIA patients (31) and 17 non-HLA variants were found to be statistically significant for a diverse range of clinical outcomes, such as actively inflamed joints and joints with limited range of motion, in a Nordic cohort of 193 patients of all subtypes excluding sJIA (15). In Kawasaki disease (KD), *NEBL* (OR 32.22) and *TUBA3C* (OR 21.03), both associated with cardiac muscle and tubulin respectively, were recently identified as risk factors for coronary artery lesions (20). While the current first line disease-modifying anti-rheumatic drug (DMARD) for all JIA subtypes remains as methotrexate (MTX), its limited efficacy necessitates the accurate prediction of MTX responders so that second-line therapy can be instituted in good time to those who do not to prevent disease progression. A 2014 study involving a cohort of 759 JIA patients from the United Kingdom, the Netherlands and Czech Republic surprisingly did not demonstrate significant association between the MTX pathway genes and treatment response, but instead identified other loci such as those related to TGF-β signaling as novel pathways for MTX response (16). Future targeted replication of these regions thus facilitates the optimization of genetic risk models for MTX response prediction.

### Sequencing of the TCR Repertoire

T-cell receptor (TCR) sequencing is targeted toward the complementary determining region 3 (CDR3) loops, where most of the diversity in these heterodimeric cell-surface receptors is contained. The CDR3 regions are formed by random rearrangements between noncontiguous variables, diversity and joining (VDJ) gene segments in the β-chain locus, and between analogous variable and joining (VJ) gene segments in the α-chain locus (32). This process drives the generation of a diverse array of TCRs, with each T-cell clonotype possessing a specific TCR, which permits the adaptive immune system to recognize cognate antigens and mount an immune response. As pathogenic derangements in T-cell biology are highly implicated in the breakdown of immunologic self-tolerance integral to the development of pediatric rheumatic diseases such as JIA (33), characterization of the TCR repertoire thus provides an additional avenue on top of conventional immunophenotyping to understand disease pathogenesis, prognosis as well as response to treatment (Table 1). ImmunoSeq, a well-established technique that is developed by Adaptive Biotechnologies, uses a multiplex PCR and sequencing approach based on a synthetic immune receptor repertoire that minimizes amplification biases (34).

TCR sequencing has helped to reaffirm trafficking of CD4 subsets shared between the autoimmune synovial microenvironment and the systemic circulation in JIA patients (22, 23). Circulating pathogenic-like lymphocytes (CPLs), a subset of circulatory CD4 T effector (Teff) cells that mirror the pro-inflammatory phenotype of synovial CD4 T cells and expressing HLA-DR, were identified in significantly greater numbers in patients with active JIA who were resistant to methotrexate (MTX) and anti-TNF-α therapy (22). Notably, the TCR repertoire of these CPLs were highly enriched in synovial clonotypes, indicating the trafficking of these pathogenic cells to or from the synovial microenvironment. While it still remains unclear whether CPLs provide the autoimmune insult or have recirculated following activation in the inflamed synovium, a direct link between CPLs and disease activity has nevertheless been established with CPLs surfacing as a plausible marker for monitoring disease activity and treatment response. A similar dysregulation was also recognized in the regulatory T (Treg) compartment, whereby a subset of Treg cells defined by HLA-DR was enriched in active JIA patients (23). TCR sequencing indicates these inflammation-associated Treg (iaTreg) cells cosegregated with synovial Treg cells rather than with other blood Treg cells, and a small fraction of iaTreg clonotypes was found to demonstrate partial overlap in TCR repertoire with arthritis-associated synovial Teff cells and blood CPLs. This hints at the importance of the inflammatory milieu which exerts an antigenic selection force in shaping systemic immunological processes. Therefore, TCR sequencing has pinpointed accessible diagnostic reservoirs of pathogenic cells that are likely to have recirculated into the bloodstream and correlated to disease activity. This paves the way to diagnostics that will prove to be a major improvement from current disease scoring systems (35), whose reliance on clinical signs and blood proxy inflammation parameters (e.g., ESR) are largely limited in accurately assessing disease progress and treatment response.

In JIA patients non-responsive to conventional DMARDs or biologics therapy, immune reconstitution through autologous hematopoietic stem cell transplantation (HSCT) may be one of the remaining options (36). Two illustrative studies have indicated TCR repertoire restriction in the Treg compartment (24, 25) of JIA or JDM patients prior to HSCT as compared to healthy controls. This points to a strong disease antigenic driving stimulus, and in particular patients who remain in remission with HSCT, had their TCR diversity restored as compared to relapse patients who retain a restricted oligoclonal profile (24). In a separate study, dominant TCR clones prior to transplantation were partially but not completely eliminated in remission sJIA patients, but rather restoration of TCR diversity suffices (26). Understanding on the mechanism of TCR diversity in relation to disease remission and its therapeutic implications has yet to be fully addressed.

## Transcriptomics

The transcriptome is the entire composite set of transcripts, both coding and non-coding, usually retrieved from a pre-selected subset of cells at a particular instance. This selective combination of transcripts, or the expression profile, gives another layer of biological insight pertaining to gene function, interaction and regulatory networks, which otherwise may not be apparent from the entire genetic sequence. From microarrays that capture limited ranges of known messenger RNAs (mRNAs) to the high-throughput next-generation sequencing (NGS) that can interrogate massive amounts of RNA in a genome-wide fashion, transcriptomics has greatly complemented genetic studies by identifying gene expression signatures for diagnostic discrimination or for shedding light on disease mechanisms (37), Table 2.

**Table 2:** Summary of recent immunomics applications and their impact on our understanding of pediatric rheumatic disease (II).

**Immunomics techniques**	**Clinical application and discovery**	**References**
**Transcriptomics**		
Microarrays	cSLE disease activity and realization of innate immunity as part of immunopathogenesis	
	Type I interferon signature and type I interferon-inducible gene expression	(38–40)
	JIA pathogenesis and treatment	
	Dysregulated interleukin-1 pathway in sJIA with active disease, anti-IL 1 therapies were introduced with good outcomes	(41–45)
	Differences in PBMC transcriptomics profiles – subtype-specific and/or disease state-specific in sJIA and non-sJIA	(39, 46–50)
	Neutrophil-specific transcriptional abnormalities persist in polyarticular JIA irrespective of disease state, suggesting aberrations in neutrophil metabolism	(51, 52)
	Kawasaki disease diagnosis	
	Whole blood gene expression signature – separates the disease from other childhood febrile illnesses	(53)
MicroRNA (miRNA)	JDM disease activity	
	Downregulation of miRNA-10a associated with increased expression of NF-_*k*_B-controlled inflammatory mediators	(54)
RNA sequencing (RNA-seq)	sJIA disease activity	
	NK cell gene dysregulation (increased expression of innate genes *S100A9* and *TLR4*, decreased expression of immune-regulating genes *IL10RA* and *GZMK*) in active disease	(55)
	JIA pathogenesis	
	Increased autophagy with up regulation of two key genes, fatty acid synthase (*FASN*) and carnitine palmitoyltransferase 1A (*CPT1A*) within the fatty acid synthesis pathway	(56, 57)
	JIA treatment response	
	Monocyte gene expression profile may predict methotrexate non-responders	(58)

*JIA, Juvenile Idiopathic Arthritis; sJIA, systemic JIA; JDM, Juvenile Dermatomyositis; PBMC, Peripheral Blood mononuclear Cells; SLE, Systemic Lupus Erythematosus; TLR, Toll-like receptor*.

### Microarrays

Microarrays quantitatively measure mRNA levels for thousands of genes in a biological sample, by relying on collections of oligonucleotide probes that capture cDNA or antisense RNA under high stringency conditions. Immobilized in defined positions on a solid matrix, labeled single-stranded nucleic acid fragments can be hybridized to these probes, and the amount of hybridization detected for a particular probe is proportional to the number of complementary fragments in the sample. Advances over the past decade have led to arrays for analysis of gene regulation (e.g., detecting microRNA), genome methylation signatures and even individual exons to assess alternative splicing. Due to their significantly greater dynamic range than reverse transcription polymerase chain reaction (RT-PCR) assays, microarrays are hence more adaptable to genome-wide high-throughput studies integral to decrypting the complex genetic networks in pediatric rheumatic disease (Table 2).

Since the 2000s, transcriptomic profiling of peripheral blood cells via microarrays has resulted in major discoveries in processes driving pediatric rheumatic diseases. In 2003, several groups independently identified the type I interferon signature in both pediatric and adult SLE patients (38–40). In particular, the set of type I interferon-inducible genes was remarkably homogeneous amongst 28 out of 29 active pediatric SLE patients who were of different ethnicities and had exhibited varying degrees of disease activity (39). As such, these findings resulted in a paradigm shift to recognize the importance of innate immunity in pediatric SLE, which was contrary to the prevailing consensus that focused on adaptive immunity stemming from the disease's characteristic autoantibody production. This has subsequently encouraged similar studies in other rheumatic diseases such as dermatomyositis, systemic sclerosis and rheumatoid arthritis (59–61), with the partial overlap in interferon signatures suggesting commonalities in disease pathophysiology (62). Microarray analysis in sJIA patients also uncovered the role of the dysregulated interleukin-1 pathway in sJIA pathogenesis, whereby the interleukin-1 signature was most pronounced in patients with systemically active sJIA (41, 42). Such work has led to the development of therapies that specifically target the offending cytokines: several type I interferon therapeutics for use in SLE (e.g., anti-IFNα monoclonal antibodies [mAb] sifalimumab and rontalizumab, anti- IFNα/β receptor mAb anifrolumab) have undergone clinical trials over recent years with reasonable reduction in disease activity and normalization of cytokine signatures, albeit trial cohorts that consisted mainly of adult patients (63–65). Similarly, several IL-1 inhibitors (e.g., IL-1 receptor antagonist: anakinra, anti-IL-1 fusion protein: rilonacept, anti-IL-1beta mAb: canalikumab) have also proven to be considerably efficacious in clinical trials that recruited patients with long-lasting sJIA and poor response to DMARDs and biologics (43–45). In particular, anakinra has entered clinical practice with favorable outcomes noted in sJIA patients, especially when started early in the disease course with or without concomitant glucocorticoids (66–68).

The late 2000s saw several studies that sought to define disease-specific signatures as well as the biological basis behind various clinical phenotypes, especially active disease versus clinical remission. A 2007 study found 286 genes that were significantly up-regulated in peripheral blood mononuclear cells (PBMCs) isolated from active sJIA patients, and this signature was proposed to be disease-specific as most of the candidate genes did not overlap with those identified for other inflammatory diseases including RF-negative polyarticular JIA, KD and pediatric SLE (39, 46–48). Distinct gene expression profiles in PBMCs that segregate active and inactive sJIA were also identified, though results may be confounded by differences in treatment regimens (48). A separate analysis pinpointed subtype-specific transcriptomic profiles in PBMCs of treatment-naïve JIA patients, particularly between sJIA and non-sJIA subtypes (49), and a contemporary study on RF-negative polyarticular JIA revealed considerable heterogeneity between gene expression patterns of PBMCs at different disease states (active, clinical remission on/off medication) (50). Evidence for chronic neutrophil activation in RF-negative polyarticular JIA (51) led to a dedicated effort to characterize the gene expression profiles of neutrophils at different stages of the disease (52). Of note, neutrophils isolated from patients with inactive disease exhibited specific transcriptional abnormalities that fail to return to normal and were linked to aberrations in neutrophil metabolism (52).

Recent work has looked into whole blood gene expression profiles to determine diagnostic biomarkers as well as predictors of treatment response, in spite of the inherent “noise” from the composite signatures of multiple cellular subsets. The relative ease of collecting whole blood as opposed to fractionated cell subsets and the holistic examination of cross-talk between all innate and adaptive immune cells in disease pathogenesis make whole blood nevertheless an attractive candidate. Using whole blood microarray gene expression data obtained from the Trial of Early Aggressive Therapy in JIA (TREAT; ClinicalTrials.gov registry #NCT00443430), network approaches utilizing functional co-expressing gene modules unveiled extensive re-ordering of gene expression networks in polyarticular JIA patients following initiation of therapy. In particular, distinctions existed between responders and non-responders on how these networks evolved (69). A follow-up study compared whole blood gene expression data between TREAT subjects at baseline, a treatment-naïve independent cohort as well as healthy controls (70). One hundred and fifty-eight genes showed differential expression with at least a 1.4-fold difference (false discovery rate 0.05) when TREAT subjects were contrasted with healthy controls, with particular enrichment of genes regulating leukocyte adhesion and extravasation (especially interleukin-8) as well as CD3-TCR signaling. In the same study, a multi-omics approach combining GWAS and microarray expression data surprisingly found that none of the 158 genes were located within linkage disequilibrium blocks containing JIA-associated SNPs, which proposes the role of the non-coding genome in JIA pathogenesis (70). A 2018 study uncovered a 13-transcript whole blood gene expression signature (of which 7 were connected in a central hub of tumor necrosis factor and interleukin 6) that distinguished KD in the first week of illness from other febrile conditions (e.g. staphylococcal and streptococcal toxic shock syndromes, measles and other viral illnesses as well as childhood inflammatory diseases)(53). This signature displayed reasonably high specificity and sensitivity for early diagnosis (discovery: sensitivity 81.7%, specificity 92.1%; validation: sensitivity 85.9%, specificity 89.1%), with predictive performance in patients with definite, highly probable or possible KD in the validation set mirroring certainty of clinical diagnosis (area under curve [AUC] 98.1% 96.3% and 70.0% respectively).

There have also been contemporary microarray studies outlining the role of microRNAs (miRNAs) and individual exons in the pathophysiology of pediatric rheumatic diseases. miRNAs are short non-coding RNA molecules that downregulate specific mRNA transcripts either by translational repression or mRNA cleavage (71), and they have been shown to alter gene expression and signaling in immunological processes as well as autoimmunity (72–74). Based on evidence implicating several miRNA species in adult inflammatory myopathies (e.g., dermatomyositis and polymyositis) (75, 76), miRNA expression in muscle biopsies isolated from 15 children with active untreated confirmed or probable JDM was compared with that of 5 healthy controls (54). miRNA-10a was found to be significantly downregulated by −2.27-fold, which was in turn associated with increased expression of NF-kB-controlled inflammatory mediators (e.g., IL-6, IL-8, TNF-alpha) as well as clinical and laboratory features of JDM (serum von Willebrand factor antigen level, Disease Activity Scores). Furthermore, miRNA and exon microarrays revealed distinct miRNA and gene isoform expression profiles in neutrophils from patients with active untreated RF-negative polyarticular JIA, though considerable overlap was noted in children with cystic fibrosis that is also characterized by chronic soft tissue inflammation (77). While the two phenotypes also shared several miRNAs and genes in their networks and annotated functions, hub miRNA networks remained unique to each disease. Future work of how the transcriptome and regulatory networks change in response to therapy may hence potentially unravel underlying disease pathogenesis to enable future rationalization of therapy.

### RNA Sequencing

RNA sequencing (RNA-seq) with NGS is the direct ultra-high-throughput sequencing of cDNA derived from transcripts in the sample, and it has several considerable advantages over microarrays: (a) detection of transcripts is free from probe-specific hybridization thus avoiding the need for a priori knowledge of targets, (b) broader dynamic range, (c) lower background signal, (d) increased sensitivity and reproducibility. As such, RNA-seq is able to efficiently measure genome-wide RNA abundance, detect novel and/or allele-specific transcripts and pinpoint alternative splice variants associated with pediatric rheumatic disease in an unbiased fashion. With that, RNA-seq is better poised as a discovery platform for holistic deep expression analysis as compared with microarray systems which is typically customized for specific questions (Table 2).

Recent comprehensive RNA-seq transcriptome analyses, particularly in combination with fluorescence-activated cell sorting (FACS) or magnetic sorted cells have aided in the identification and characterization of dysregulated pathways in disease implicated cellular subsets. As sJIA involves prominent innate immune activation and lacks significant involvement of autoreactive T cells or autoantibodies (29), recent studies have used RNA-seq to define mechanistic, diagnostic, and predictive signatures in specific innate immune cells. Genome-wide RNA sequencing revealed 214 differentially expressed genes in magnetically sorted neutrophils from the blood of children with active sJIA disease compared with healthy controls (78). The most significantly upregulated gene pathway in active sJIA disease corresponded to “Immune System Process” including genes such as *AIM2, IL18RAP, NLRC4*, and IL-18 expression remains dysregulated at a lower intermediate level even in clinically inactive state as compared with healthy individuals. Another study sought to delineate the role of natural killer (NK) cells in sJIA by performing comparative RNA sequencing analysis of these FACS sorted NK cells from a cohort of 6 active sJIA patients and 6 healthy controls (55). Proinflammatory mediators IL-1β and IL-6 were identified to be major upstream drivers of NK cell gene dysregulation (e.g., increased expression of innate genes *S100A9* and *TLR4*, decreased expression of immune-regulating genes *IL10RA* and *GZMK*) in active disease. In conjunction with an altered plasma cytokine profile enriched in species that promote inflammation and NK cell survival, this thereby implicates the inflammatory milieu characteristic of sJIA in shaping the biologic behavior of NK cells and their consequent function in disease pathogenesis. Moving forward, future work can hence aim to better define RNA signatures in cells of innate immunity in sJIA patients that is stratified in relation to therapy response.

In non-systemic JIA subtypes, studies have focused on the adaptive immune system, whose role in disease pathogenesis has been well-documented. Transcriptomic analyses of sorted CD4^+^ synovial T cells of patients with active disease (13 oligoarticular, 8 extended oligoarticular, 3 polyarticular) demonstrated enhanced expression of autophagy genes compared with PBMCs of patients and healthy controls (56). Interestingly, this was not accompanied by significant upregulation of autophagy in the presence of synovial fluid, yet the inflammatory phenotype of these cells was impaired on inhibition of autophagy with hydroxychloroquine. As autophagy is a cell-survival mechanism that permits energy and nutrient conservation (79), it was hence postulated that the increase in autophagy may have occurred to cope with the greater metabolic demand of inflammation, and targeting autophagy in dysregulated T cells may be a viable strategy to restore disrupted T cell homeostasis in JIA. Indeed, a separate study indicate that sorted CD4^+^ memory T cells in RA/JIA patients exhibit higher autophagy, termed as “autophagic memory,” that affords for better persistence through this metabolic advantage (57). This phenomenon was shown through transcription factor gene regulatory network analysis (TF-GRN) of the transcriptome (RNA-seq) of sorted JIA pathogenic T cells (CPLs), to be driven by the suppression of the *MYC* gene (57). Furthermore, RNA-seq data from CPLs indicate the up-regulation of two key genes, fatty acid synthase (*FASN*) and carnitine palmitoyltransferase 1A (*CPT1A*) within the fatty acid synthesis pathway (57), adding weight to the idea of metabolic advantage.

While the role of innate immunity in non-systemic JIA subtypes is less clear, emerging evidence has hypothesized the importance of neutrophils in linking both arms of the immune system in disease pathogenesis. Notably, prior microarray analyses reflected differences in neutrophil expression profile that correlated with disease phenotypes in RF-negative polyarticular JIA (52). The same authors sought to substantiate those findings by investigating the transcriptomes of neutrophils from 9 individuals (3 with active untreated RF-negative polyarticular JIA, 3 with the same disease that was inactive on medication, 3 children with cystic fibrosis) (80). One hundred and fifty nine genes were differentially expressed in children with active disease when compared to those with sustained inactive disease on medication (e.g., downregulation of type I interferon response genes and interferon-induced proteins in active disease), while 113 genes showed differential expression with at least 1.9-fold change (*p* < 0.05) when neutrophils from children with untreated RF-negative polyarticular JIA were compared with those from children with cystic fibrosis. Differential exon usage genes and long non-coding RNA (lncRNA) expression were also identified between the disease phenotypes. Interestingly, the prior study on sJIA reported a dissimilar neutrophil gene expression signature that lacked significant upregulation of IL-8 and IFNγ. Though there is a need to evaluate these findings in larger cohorts, both studies further contribute to the promise of potential neutrophil biomarkers for diagnosis and prognosis across JIA subtypes, given the proposed adaptability in neutrophil transcriptomes under specific biological contexts.

There have been studies in non-systemic JIA subtypes that chose instead to work with unfractionated heterogeneous cell populations (e.g., PBMCs), though differing outcomes were noted in biomarker identification. Analysis of gene expression patterns in PBMCs of patients with polyarticular JIA at different treatment stages (active untreated disease, active on treatment or clinical remission on medication) as well as with healthy controls surprisingly failed to define molecular signatures that would assist in disease staging (active disease vs. clinical remission) or in diagnosis (untreated active disease vs. healthy controls) (81). In retrospect, the authors attributed this challenge to technical issues in RNA-seq and biological factors stemming from the heterogeneity within polyarticular JIA as well as PBMCs. On the other hand, examining the transcriptomic profile of PBMCs in oligoarticular and polyarticular JIA prior to MTX has yielded promising results (58). In this cohort that possessed an MTX response rate of 61.7% as defined by the ACR-Ped criteria, a signature predictive of eventual response was elucidated from 47 patients whose clinical outcomes were measured pre- and at least 2 months post-MTX treatment. The gene expression profile of MTX responders was distinct from, but more similar, to healthy controls than that of non-responders. There was also a strong correlation between the mean MTX non-responder signature with monocyte gene expression, which suggests the potential role of innate immunity in clinical response to MTX. While technical and bioinformatics noise remain as considerable issues especially in dealing with unfractionated cell populations in poorly-defined diseases, future work in improving library preparation and spike-in controls as well as developing appropriate computational approaches for data post-processing will hopefully augment our use of this powerful technology to understand disease mechanisms.

## Epigenomics

Epigenomics broadly entails the hereditary and phenotypic traits that can alter function at the genome level without a direct change in the genetic sequence (82). These epigenetic mechanisms allow for genetic and environmental factors to interact and contribute to particular phenotypes and diseases. There has been growing evidence to suggest that epigenetic modifications are implicated in several autoimmune diseases, e.g., modifications to DNA methylation has been detected in SLE, RA and Type 1 diabetes mellitus (83). Discovery of these epigenetic changes will provide another layer of dimension toward understanding how disease mechanisms operate holistically and ultimately allow for biomarker development for prognostic and diagnostic applications (Table 3).

**Table 3:** Summary of recent immunomics applications and their impact on our understanding of pediatric rheumatic disease (III).

**Immunomics techniques**	**Clinical application and discovery**	**References**
**Epigenomics**		
CpG DNA Methylation	sJIA disease activity	
	CD4+ T cell DNA methylation was significantly decreased at the IL-32 gene as compared to healthy controls	(84)
	Certain CpG modules were statistically related to clinical fates and enriched on genes responsible for T cell activation in JIA patients with active disease before and after withdrawal of therapy	(23)
	Kawasaki disease pathogenesis and treatment	
	Hypomethylation within the promoters of TLR1, 2, 4, 6, 8, and 9 (whole blood), correlated with increase in mRNA expression of respective TLRs compared to healthy and other febrile controls, and reversed upon treatment with intravenous immunoglobulin (IVIG)	(85)
	DNA hypomethylation (whole blood) of *FCGR2A* was associated with resistance to intravenous immunoglobulin (IVIG) treatment	(86, 87)
Chromatin immunoprecipitation (ChIP) Assays	JIA pathogenesis	
	In JIA patients, neutrophils and CD4+ T cells exhibited H3K4me1 and/or H3K27ac marks in the non-coding areas of genetic risk, suggesting the crucial role of non-coding elements within leukocyte genomes	(88)

*JIA, Juvenile Idiopathic Arthritis, TLR, Toll-like Receptor*.

### CpG DNA Methylation

DNA methylation of gene promoters, specifically at regions of CpG dinucleotides, is usually associated with reduced gene expression (89). Numerous studies conducted for adult rheumatological diseases have implicated aberrant DNA methylation (83). In RA, DNA methylation was examined specifically through bisulfite sequencing at a loci containing 22 CpG motifs upstream of the IL-6 gene (90). The reduction in DNA methylation in the−1099CpG motif was in tandem with an increased expression of IL-6; that is in line with the pathophysiology of RA, a chronic inflammatory disorder.

Technologies to examine DNA methylation have undergone an increase in their capacity to examine unique CpG sites upon the creation of arrays in the late 1990s. For instance, current DNA methylation array platforms such as the Illumina Infinium HumanMethylation450 (M450K) BeadChip are now able to target more than 450,000 methylation sites, giving us a genomic wide view of epigenetic disruptions. This epigenomic view through CpG arrays is illustrated in a study on how differential T-cell DNA methylation may impact JIA (91). Before this study, there were no prior studies about epigenetic disturbances in JIA. As epigenetic marks may be amenable to modification and thus serve as candidate therapeutic targets (84), they sought to profile DNA methylation of purified CD4^+^ T cells from healthy controls and JIA subjects. The Illumina platform was used to compare more than 25,000 CpGs sites, and analysis found significant decreased methylation at the IL-32 gene.

In a recent study, a genome-scale case-control analysis of CD4^+^ T cell DNA methylation in oligoarticular JIA was conducted (92). The Illumina HumanMethylation 450 array was deployed to examine DNA methylation of >450,000 sites in sorted CD4^+^ T cells from JIA patients. However, in contrast to the earlier JIA study as well as other adult-onset rheumatic diseases such as RA and SLE (93), the authors found no significant differences in the DNA methylation profiles between disease and controls. The authors attribute the differences between the studies to the targeted selection of CD4^+^ T cells in one particular subgroup (oligoarticular) of JIA. However, independently in another study, the CD4^+^ T cell DNA methylome of both polyarticular and extended oligoarticular JIA patients prior to and after withdrawal of antiTNFα therapy was investigated (23), to answer a pertinent clinical need in segregating patients who have either resolved disease or still require constant therapy. To allow for better noise reduction, CpG sites were analyzed with weighted gene co-expression network analysis (WGCNA), which clustered the CpG sites into statistically correlated CpG modules that are likely to be biologically correlated (23). In particular, this study revealed that certain CpG modules were statistically related to clinical fates, where JIA patients who are active prior/after withdrawal of therapy, were enriched for genes responsible for T cell activation.

Yet some studies have looked at a mixture of cell types to characterize biological differences with the progression of clinical treatment. One particular study on KD patients, examined the entire white blood population with the Illumina M450K beadchip, in an attempt to identify patterns of DNA methylation of all 10 human toll-like receptors (TLRs), typically known to be expressed across several cell types (85). The CpG sites within the promoters of TLR1, 2, 4, 6, 8, and 9 were hypomethylated in KD patients, and this was in line with the increase in mRNA expression of the respective TLRs. This was shown to be true when comparing the KD patients against the healthy or febrile non-KD controls, and the trend reversed upon treatment with intravenous immunoglobulin (IVIG) in KD patients. The reversal in CpG hypomethylation comes as a surprise, as epigenetic modifications tend to be stable, so this reversion could likely have resulted from a change in cellular frequency of certain immune subsets during the course of treatment. Nonetheless, studies have shown that TLRs 2, 3, 4, 6, and 9 may be the initial triggers for the immune response in KD patients (94), thus suggesting that epigenetic predisposition in TLRs (or dysregulation in specific immune subsets) may “sensitize” KD patients and play a crucial role in disease risk and pathogenesis. The same group also revealed a positive association between DNA hypomethylation of *FCGR2A* and resistance to IVIG treatment in KD patients (86, 87). The *FCGR2A* gene codes for the low-affinity immunoglobulin gamma Fc region receptor II-a protein, expressed on a variety of immune subsets and in particular phagocytes such as monocytes and macrophages. Pyrosequencing reconfirmed that patients who were IVIG-resistant had significantly lower *FCGR2A* methylation levels at all 5 CpG methylation sites studied than those who were IVIG-responsive (86). This significant hypomethylation was accompanied by significantly higher *FCGR2A* mRNA levels in KD patients compared to febrile controls. The clinical relevance was later determined (87): hypomethylation of the CpG marker cg24422489 at the *FCGR2A* gene promoter in KD patients was reversed after IVIG was administered, with a concomitant increase in *FCGR2A* mRNA expression. The authors suggest that *FCGR2A* likely play a pro-inflammatory role with increased susceptibility to KD and thus may provide a mechanistic rationale for the usage of IVIG in KD. These studies provide an additional layer of biological insight into how epigenetic mechanisms and their candidate target genes can influence disease pathology and treatment response.

### Chromatin Immunoprecipitation (ChIP) Assays

ChIP is an immunoprecipitation technique that enables analysis of a spectrum of protein-DNA interactions, including transcription initiation factors on promoters or silencers on regulatory sites as well as the specific localization of defined histone modifications (95). This is performed with the intent to identify the DNA sequence to which a specified target protein complex binds either directly to or in a chromatin folded conformation. A variation of this technique is ChIP sequencing (ChIP-seq), which is able to identify DNA binding sites more precisely. In ChIP sequencing, oligonucleotide adaptors are added to the DNA bound to the target protein of interest and subsequently sequenced (95).

ChIP has been used in large-scale studies, namely the Encyclopedia of Functional DNA elements (ENCODE) and Roadmap Epigenomics projects. DNA-binding proteins such as enhancers and silencers cannot be predicted accurately *in-silico*, solely based on the DNA sequence (88). ChIP-seq plays a vital role in validating this physical interaction. The ENCODE and Roadmap Epigenomics projects showed that using ChIP-seq to direct to particular histone marks such as histone H3 mono-methylated at lysine 4 (H3K4me1) (96) can facilitate the identification of enhancers.

In pediatric rheumatic diseases, ChIP assays have been used in JIA, typically followed by sequencing (Table 3). Jiang et al. (88) used ChIP-seq to find out if there are specific epigenetic marks (H3K4me1and H3K27ac) associated with enhancer function in human neutrophils and CD4^+^ cells (88). This was a follow-up from a GWAS study that showed 24 regions (or SNPs) of genetic risk for JIA, of which 22 were in noncoding genomic regions (14). The aim was to determine if there were functional elements situated in these non-coding areas of genetic risk. ChIP-seq was specifically used to check for enhancer-associated histone marks within the linkage disequilibrium blocks that comprises the 22 regions found via the GWAS. It was found that these linkage disequilibrium blocks are indeed rich in histone marks commonly associated with enhancers, adding further weight on the disease susceptibility risk loci previously identified in GWAS.

Separately, Peeters et al. (97) used H3K27ac to identify a typical enhancer and super-enhancer signature in the CD4^+^ memory and effector T cells derived from the synovial fluid of JIA patients (97). Use of the BET (bromodomain and extra-terminal domain) inhibitor JQ1 was found to inhibit super-enhancers that are related to immune response, in addition to reducing disease-associated gene expression. BET inhibitions have been previously shown to preferentially reduce super enhancer-associated gene expression (98). These results are specific to the synovial microenvironment and suggest that enhancer profiling could be used for the identification of disease mediators. BET inhibition can also be explored as a potential therapeutic for autoimmune disease treatment.

## Proteomics

Proteomics refers to the large-scale study of the entire complement of proteins and strives to understand the expression profiles, interactions, and functions of these proteins (99). What makes this landscape so complex is the enormous permutations to which proteins can be differentially expressed (splice forms) or modified, with their spatially and temporally distinct formats, culminating in a complex diversity of interactions. Proteins are deeply involved in the manifestation of cellular phenotypes, and the study of proteins can present succinct clues on immune cellular behavior and function.

### Mass Spectrometry

Mass spectrometry (MS) became the predominant technique for examining proteins (100) at a proteome level with technological advances in particular to mass selection, detection, and analysis (101) gradually taking form. MS facilitates the acquisition of protein information, including protein identity (amino acid sequences), abundance and post translational modifications through accurate assessment of atomic mass spectra. There are three generic stages involved in the procurement of protein information by MS: sample preparation, sample ionization, and mass analysis (100, 101). Frequently, before a complex protein mixture can be analyzed by MS, it has to be resolved (e.g., trypsin digestion) and extracted using chromatographic means (e.g., reverse phase or pH). The resulting peptides have to be charged through soft ionization techniques (e.g., MALDI or ESI) and desolvated, prior to passing through mass filtering by designated quadrupoles and finally undergo mass analysis by detectors (e.g., orbitrap or time of flight). The acquired data (MS^1^ and/or MS^2^ spectra) is cross referenced against a mass spectra database through a software designed for the mass spectrometer configuration.

MS platforms are now adept at distilling candidate protein targets and increasingly being deployed to characterize proteomic profiles of pediatric rheumatic diseases (Table 4). One study found that different systemic autoimmune diseases (SAID), including JIA and JDM, share similar dysregulation in plasma protein expression and affected pathways (108). To reduce background noise from polymorphic genes, matched monozygotic twins that are discordant for disease development were studied, and plasma proteins found significantly different from the twins were further compared against other matched unrelated controls. Plasma protein levels were examined using liquid chromatography-electrospray ionization-tandem mass spectrometry (LC-ESI-MS). Pathway analysis revealed significant dysregulation in acute phase reactants, complement pathway, coagulation and retinoid receptor activation in SAID patients. With the aid of random forest modeling, 7 top proteins were identified, that were interconnected through paraoxonase 1 and a secondary link to IL-6, thus providing a candidate list of afflicted proteins and pathways present in SAID patients.

**Table 4:** Summary of recent immunomics applications and their impact on our understanding of pediatric rheumatic disease (IV).

**Immunomics techniques**	**Clinical application and discovery**	**References**
**Proteomics**		
Mass spectrometry	JIA classification	
	Distinct proteome profiles between the subgroups (oligoarticular and polyarticular) in early disease	(102)
	Polyarticular JIA patients expressed higher levels of platelet activation factors, including fibrinogen-β/γ chains	(103)
	Type VI collagen was found at higher levels in oligoarthritis patients	(104)
	Childhood-onset SLE with nephritis - biomarkers for disease activity	
	Eight stable urinary proteomic signatures present in patients with nephritis, displayed a strong correlation with renal disease and moderate correlation with renal damage	(105)
Multiplex enzyme-linked immunosorbent assay (ELISA)	JIA with methotrexate response	
	Predominant cytokine clusters during active/inactive disease were identified - several cytokines such as CCL2, CCL3 and CXCL9 were found to be significantly increased in the plasma of JIA patients, coinciding with inflammation	(106)
	JDM disease activity monitoring	
	CXCL10, TNF receptor Type II and galectin 9 showed significant increases in active JDM and strongly correlated to active disease and clinical JDM scores	(107)

*JIA, Juvenile Idiopathic Arthritis; JDM, Juvenile Dermatomyositis; SLE, Systemic Lupus Erythematosus*.

In JIA, MS has been employed to differentiate clinical subtypes. With the aid of MALDI-TOF-MS, Finnegan et al. (102) studied 15 treatment naive JIA patients and found distinct proteome profiles between the subgroups (oligoarthritis and polyarthritis) during early disease (102). The group found significant differences in expression levels of proteins involved in coagulation and platelet activation. Polyarticular JIA patients, who exhibit a more severe clinical presentation, expressed higher levels of fibrinogen-β/γ chains known to mediate polymerization of fibrin and binding to thrombin (103). Pathological changes in coagulation pathway proteins may contribute to the inflammatory spread across joints, which is observed in polyarticular JIA patients (102). In contrast, Type VI collagen was found at higher levels within oligoarticular JIA patients. Type VI collagen is known to be crucial for regulating normal synovial joint physiology where mice lacking collagen Type VI had a significant reduction in mechanical properties and experienced a myriad of musculoskeletal issues (104).

Apart from distinguishing disease subtypes, proteomics profiles have been exploited for biomarker discovery. Surface-enhanced laser desorption/ionization time-of flight (SELDI-TOF) MS was used in one study (105) of pediatric SLE patients, allowing for high-throughput profiling of urine samples and sensitive detection of low-molecular-weight biomarkers that may be missed by other conventional methods (109). A stable urinary proteomic signature encompassing eight proteins was indicative for pediatric SLE patients with nephritis. These markers displayed a strong correlation with renal disease and moderate correlation with renal damage. Identification of this urine proteomic signature may help in prediction of SLE renal disease prior to nephritis presentation.

### Multiplex Enzyme-Linked Immunosorbent Assay (ELISA)

The enzyme-linked immunosorbent assay (ELISA) is commonly used for profiling of selected liquid analytes, in particular pertaining to immunological response. It uses an enzyme immunoassay (EIA), i.e., an enzyme reaction with its substrate, to detect the presence of a target antigen using specific antibodies. In pediatric rheumatic diseases, this technology has been actively used to profile cytokines or mediators involved in the disease process (Table 4). For example in JIA, decreased production of IL-10, a regulatory cytokine, has been found to be accompanied by increased pro-inflammatory cytokines (110). Conventional singleplex ELISA kits provide only a singular snapshot of the selected immune mediators, eventually increased in interrogation spectrum. With the development of beads or particle based multiplex immunoassays (MIAs). Current MIA kits (e.g., Luminex) allows the simultaneous detection of up to 65 unique mediators from samples in microliter volume. One MIA was developed for the detection of 30 inflammation-related human soluble mediators in plasma and synovial fluid, specifically in JIA (106). Using this assay, they were able to measure a diverse panel of chemokines, interleukins (ILs), and soluble adhesion molecule to create biochemical profiles for healthy controls and JIA patients. Cluster analysis of these results showed differences between active disease and remission. There was a predominant pro-inflammatory cytokine cluster during active disease, in contrast to an anti-inflammatory-related cytokine cluster during remission. Several cytokines such as CCL2, CCL3, and CXCL9 were found to be significantly increased in the plasma of JIA patients coinciding with active inflammation. MIAs have also been used in JDM to find markers for disease activity monitoring. This would allow for better personalization of therapeutic regimens. In one particular study that looked at 45 unique inflammation-related proteins in 25 JDM patients, 3 proteins were significantly elevated compared to the control group (107). CXCL10, tumor necrosis factor receptor Type II and galectin 9 displayed significant increases in active JDM. These were also strongly correlated to active disease and clinical JDM scores, that allows for tracking disease progression.

Cytokine profiles screened through MIA kits, could potentially be used to monitor disease activity, determine treatment response and play a role in the prediction of disease flares. Despite the relatively low-throughput in screening potential compared to mass spectrometry, the convenience and robustness in validating and deploying ELISA diagnostic kits in hospital labs, explains their utility and widespread usage.

## Cytomics

Cytomics aims to understand complex cellular landscapes and systems at the single cell level by integrating molecular techniques (e.g., dyes and fluorophores) with digital spectra acquisition. Dissection of complex immune cellular phenotypes can augment our knowledge of how disease mechanisms operate. For instance, analysis of alterations in lymphoid and myeloid cells, allow for identification of immune cell subpopulations that are disease-specific (111) and may otherwise be buried within the bulk population, Table 5.

**Table 5:** Summary of recent immunomics applications and their impact on our understanding of pediatric rheumatic disease (V).

**Immunomics techniques**	**Clinical application and discovery**	**References**
**Cytomics**		
Multi-parametric flow cytometry	Childhood-onset SLE pathogenesis	
	Impaired upregulation of PD-1 expression in monocytes and myeloid dendritic cells in active SLE patients as compared to healthy controls or SLE patients in remission, suggesting a possible mechanism in loss of peripheral tolerance	(112)
	Double negative T cell elevation (> 2.5%), in children with SLE, MCTD and ANA-positive JIA	(113)
Mass cytometry (CyTOF)	JDM pathogenesis	
	Defective phosphorylation of PLCγ2 in natural killer (NK) cells compared to healthy controls	(114)
	JIA pathogenesis	
	Treatment-naïve polyarticular JIA patients displayed enhanced IFN-γ signaling in CD4 T cells and monocytes. Naïve CD4 T cells had more strongly phosphorylated STAT1 and STAT3 as compared to monocytes, which displayed increased phosphorylation of STAT3 compared with controls. This suggests that attenuation of IFN-γ signaling could be a novel alternative therapy for polyarticular JIA.	(115)
	Childhood-onset SLE pathogenesis	
	Monocyte cytokine signatures with high monocyte chemoattractant protein-1 (MCP-1), macrophage inflammatory protein 1β (MIP1β) and interleukin-1 receptor antagonist (IL-1RA) were found in treatment-naïve SLE children	(116)

*JIA, Juvenile Idiopathic Arthritis; SLE, Systemic Lupus Erythematosus*.

### Multi-Parametric Flow Cytometry

Flow cytometry is the key platform utilized in the field of cytomics. Since its introduction more than half a century ago, fluorescence-based flow cytometry has been extensively used for functional analysis and characterization of immune cells subsets (111). Technological advancements have allowed for increasing numbers of measurable parameters per cell. The latest flow cytometers are able to detect > 20 parameters. Accompanying this increase has been the extent of targets that can be assayed. Initial flow cytometry systems were limited only to cell surface marker analysis, that eventually expanded to intra-cellular markers with cell permeabilization techniques. Now, correlation of functional cell subsets with differential kinase states can be performed with the availability of kinase specific antibodies (117). Such *in vivo* kinase assays can provide better information on signaling pathways that are crucial to understanding cellular processes and responses to receptor triggering.

Multi-parametric flow cytometry has been actively deployed in the investigation of pediatric rheumatic diseases for immune phenotyping (Table 5). As autoimmune diseases can be partially attributed to the loss of self-tolerance, investigators examined PBMCs from pediatric SLE patients with a 12 color fluorescent based panel (112). Immune phenotyping indicated a decreased capacity to upregulate PD-L1 expression in monocytes and myeloid dendritic cells in active SLE patients as compared with healthy age-matched controls or SLE patient experiencing remission, suggesting a possible mechanism in loss of peripheral tolerance (112). Independently, Tarbox et al. (113) examined the presence of double negative T (DNT) cells in pediatric rheumatic diseases, which is known to increase in autoimmune lymphoproliferative syndrome due to defects in the Fas-apoptotic pathway (113). PBMCs were analyzed by a multi-parametric flow panel (>12) from pediatric patients with SLE, mixed connective tissue disorder (MCTD), JIA and elevated antinuclear antibody (ANA) without systemic disease (113). There was a significant increase in the number of patients with DNT cells raised ≥2.5% as compared with controls. It was found that 29.6% of patients displayed elevated DNT cells, as compared to 3.6% of controls and this was stable over ~8 months, suggesting the role of DNTs/apoptosis in disease pathogenesis. Spreafico et al. (22) utilized a 12 color flow panel to allow for the sensitive detection of a circulating subset of pathogenic CD4^+^ T cells that are phenotypically similar to CD4^+^ T cells from the synovial micro-environment of JIA patients (22).These circulating pathogenic lymphocytes (CPLs) correlate significantly with disease activity and are increased in patients resistant to methotrexate and anti-TNFα therapy. As the blood serves as a convenient reservoir of cells that are easily accessible for diagnostic purposes, the authors strongly advocate the utility of tracking CPLs.

## High Dimensional Single Cell Resolution Profiling

It is increasingly clear now that the immunological landscape is complex and heterogeneous. The inability to resolve high dimensional signals at the single cell layer with conventional technologies, irrevocably result in the concealment of unique cellular signatures in bulk data (118). The emergence of single cell technologies that permit high dimensional interrogation will provide unprecedented explosion of biological data that when interfaced with clinical perspective, can present new, exciting opportunities.

### Single-Cell RNA Sequencing

Single-cell RNA sequencing (scRNA-seq) provides the transcriptome of individual cells (119), which better accounts for the stochasticity and heterogeneity in gene expression observed in populations previously thought to be similar, that now likely exist in an continuum of subsets. This is a marked improvement from traditional RNA-seq techniques that assess bulk populations and average signals from cellular populations, thereby now capturing important cell-to-cell variability that may be crucial for disease progression. In addition, scRNA-seq allows the sensitive identification of rare cell types that could have otherwise be overlooked in an analysis of pooled cells and facilitate the characterization of the spectrum of immune cell populations involved in the pathogenesis of pediatric rheumatic diseases.

Various scRNA-seq protocols have been published over the past few years, and they may be classified based on how single cells are captured and how RNA levels from a single cell is quantified. Flow-based and microfluidic technologies have been commonly used to isolate single cells: the former is ideal for selecting specific cell subsets using fluorescently-tagged monoclonal antibodies bound to specific surface markers, while the latter (e.g., Fluidigm C1) offers precise fluid control with an intricate system of valves and switches to isolate cells of interest. A modified version of conventional microfluidics technology, microdroplet-based microfluidics (e.g., 10x Genomics Chromium, Drop-seq, inDrop) allows a cell to be encapsulated in a droplet with a bead containing a unique barcode that will be attached to all downstream reads. As such, all droplets can be sequenced together in a high-throughput manner and reads accurately assigned to individual cells of origin. RNA quantification is achieved either by full-length or tag-based sequencing: while tag-based protocols only profile one end of each RNA and are hence thought to offer poorer read mappability; they are more amenable to highly parallel multiplexing and often incorporate the use of 4 to 10 bp long unique molecular identifiers that greatly reduce amplification biases (120).

The advances in scRNA-seq hardware have also spurred parallel advancements in computational handling of increasingly complex data output, which demands generic bioinformatics tools previously employed in bulk sequencing data analysis to be tailored to specific challenges in the single-cell setting. For instance, the intrinsic stochasticity amongst single cells and technical variability from transcriptome sampling result in the violation of assumptions upon which most normalization methods are based (e.g., a stable relationship between transcript count and sequence depth), and instead introduce artifacts that bias downstream analyses (121). In response, novel regression-based and machine learning approaches that consider co-variance relationships between gene expression values have been developed to deconvolve gene expression signals for normalization and discovery purposes (121, 122).

The successes of scRNA-seq in cancer biology offer many lessons due to significant parallels with pediatric rheumatic diseases, including cell type heterogeneity, complex interactions between pathogenic cells and their microenvironment as well as immune dysregulation. For example, detailed modeling of transcriptional kinetics in individual tumor cells has facilitated the study of cancer evolution. In various cancers such as those of the intestinal and hematopoietic lineages, scRNA-seq has helped to characterize the layers of tumor hierarchies to uncover cell of origins as well as previously unknown populations that may be pathologically relevant (123). scRNA-seq has also augmented, especially in the context of pancreatic cancer, the direct detection of gene expression signatures of cancer cells separately from those of infiltrating stroma, thereby permitting better characterization of the function each component plays in tumorigenesis and unraveling more specific therapeutic targets (124, 125). Future applications for scRNA-seq in cancer are hence likely to be of considerable clinical utility in providing reliable measures for risk assessment, early stage detection and monitoring of treatment response. Relating back to pediatric rheumatic diseases, scRNA-seq technologies thus show great potential for clinical translation by enabling, across disease phenotypes, unbiased characterization of distinct immune cell subsets and their accompanying stochastic variability, discovery of unidentified cell types as well as reconstruction of lineage progression.

While current literature on pediatric rheumatic diseases has yet to showcase significant scRNA-seq analyses, early successes have already been reported in the study of adult rheumatic diseases, in particular rheumatoid arthritis (RA) and adult SLE. As synovial fibroblasts play important roles in initiating and driving RA by contributing to the proinflammatory milieu and promoting osteoclast function, scRNA-seq was used as part of a toolkit to define the molecular identity of the pathogenic fibroblasts (126). Comparing synovial fibroblasts from patients with chronic late-stage RA or osteoarthritis (OA), RA-specific transcriptomic changes were noted with 3 major subsets (CD34^−^THY1^+^, CD34^−^THY1^+^, CD34^+^) identified after integrating bulk and single-cell transcriptomics. Following subsequent histological and functional assays, the CD34^−^THY1^+^ fibroblasts appeared to play the strongest role in promoting synovial swelling and inflammation. scRNA-seq has also helped to delineate SLE pathogenesis and disease complications, though studies were conducted on adult patients. For instance, transcriptomic analyses of human renal and skin biopsy samples from adult SLE patients derived a signature composed of interferon-inducible genes in renal tubular cells that correlated with clinical parameters of lupus nephritis (127). Interestingly, analysis of cumulative expression profiles of single cell keratinocytes derived from healthy non–sun-exposed skin of patients with lupus nephritis also demonstrated similar upregulation of those genes, thereby proposing the alternative use of accessible skin biopsies as a biomarker for renal disease. With concurrent advancements in sample handling ensuring reproducible downstream analysis, including a recently-published protocol verified in RA and OA for acquiring viable cells from cryopreserved synovial tissue with intact transcriptomes and cell surface phenotypes (128), tools are now in place for the profiling of human tissues for integrated analysis of immune repertoires and cell states. Moving forward, new technologies in single-cell profiling beyond transcriptomics puts forth the tantalizing prospect of multiplexing different measurements to derive a highly informative signature, thereby allowing us to better define biomarkers and therapeutic targets in pediatric rheumatic diseases.

### Mass Cytometry

The mass cytometer or CyToF (cytometry time of flight) is essentially a mass spectrometer platform designed specifically to interrogate at the single cell resolution (129), examining in excess of 40 parameters. Cells are typically examined with target specific antibodies that are conjugated to rare heavy metals (lanthanides). These heavy metals are not found endogenously in the cells, which forms the basis for relative quantification of the target parameters. In traditional flow cytometry, the emission profiles of the fluorophores overlap. The spill over from the spectral emission across channels present difficulties in precise quantification. This is commonly rectified through spectral compensation by determining the ratio of spill over but eventually limits the parameters that can be resolved. On the other hand, mass cytometry detects discrete atomic masses, which avoids the need for any compensation. Current parameter limits are determined by the number of commercially available pure heavy metal isotopes, otherwise theoretical parameter limits exceed 100. Despite these advantages, mass cytometry has its constraints. Firstly, the analytical event rate is lower than that seen in flow cytometry (111), and the agitation due to nebulization causes about 30–50% of the input cells to be lost. These cells are eradicated during the process of detection, which disallows subsequent cell sorting (130). Nonetheless, mass cytometry is a promising technological development that is well-suited to unveil the complexity of biological details (Table 5).

Mass cytometry was performed on PBMCs from treatment-native JDM patients and healthy controls in an attempt to understand cellular signaling (114). In combination with phospho-specific antibodies, the activation states of 14 signaling molecules were probed at baseline and after stimulation with cytokines and cross-linking antibodies. Defective phosphorylation of PLCγ2 in natural killer (NK) cells was the main signaling difference between patients and controls, whereby PLCγ2 hypophosphorylation was observed in patients. This PLCγ2 hypophosphorylation was correlated with reduced calcium flux via flow cytometry. Several studies implicate NK cells in JDM disease pathogenesis. NK cells are “lymphocytes” of the innate immune system and play roles in cancer surveillance and antiviral defenses (131). Studies have suggested that human NK cell dysfunction may lead to the onset of autoimmunity, and the reduced calcium flux observed in Throm et al. (114) provide insights into the downstream functional consequences.

The same group also studied signaling abnormalities in polyarticular JIA and found that treatment-naïve patients displayed enhanced IFN-γ signaling in CD4 T cells and monocytes (115). Naïve CD4 T cells had more strongly phosphorylated STAT1 and STAT3 as compared to monocytes that displayed increased phosphorylation of STAT3 in patients than controls after 15 minutes of stimulation with IFN-γ. These results suggest that attenuation of IFN-γ signaling could be a possible alternative therapy for polyarticular JIA.

Pediatric SLE patients were also studied to evaluate the presence of immune dysregulation via mass cytometry. Different studies have offered conflicting information on the involvement of specific immune cell subsets in the pathogenesis of SLE. Some studies have showed that the circulating regulatory T cells are decreased while others have shown that numbers are the same while suppression of immune response is reduced (132, 133). This could possibly be attributed to the contextual nature of how studies have focused on specific aspects of the immune system rather than examining an integrated pool of information. One study tried to offer a single-cell system-level perspective of SLE by studying newly diagnosed and treatment naïve patients (116) via mass cytometry. They found that newly diagnosed, treatment naïve SLE patients had an association with distinct monocyte cytokine signatures with high monocyte chemoattractant protein-1 (MCP1), macrophage inflammatory protein 1β (Mip1β) and interleukin-1 receptor antagonist (IL-1RA). Furthermore, these signatures were found to be inducible by plasma of active SLE subjects when the diseased plasma was incubated *ex vivo* with healthy donor's blood. This study shows the utility of mass cytometry in studying immune modifications in pediatric SLE, which may give us insights into disease pathogenesis.

## Challenges in Analysis and Sharing of High Dimensional Data

The advent of high dimensional data (e.g., in CyToF or scRNA-seq) presented unique challenges in deciphering and analysis; that saw the gradual acceptance of nonlinear dimensionality reduction algorithms such as *t*-distributed stochastic neighbor embedding algorithm (t-SNE) (134), uniform manifold approximation and projection (UMAP) (135) or refined variations of sorts (136). Application of these algorithms helps project the multi-dimensions onto a 2- or 3-dimensional space, allowing researchers to resolve and visualize high dimensional data. These studies also help provide unique insights to immune subset and pathway heterogeneity, in particular to pathological states.

Yet with the massive accumulation in high dimensional data across different platforms and experimental labs, the scientific field now faces the up-hill task of integrating diverse datasets and exploiting them for further data mining. One key initiative is ImmPort (http://www.immport.org/) (137) by the National Institute of Allergy and Infectious Diseases Division of Allergy, Immunology and Transplantation (NIAID-DAIT). As of 2018, ImmPort has amassed a depository exceeding 50,180 human/animal subjects from 1,369 experiments, spanning a variety of scientific data from CyToF, flow cytometry, serum and genetic markers or clinical variates. Uploading of data through this portal is performed through standardized templates with reproducible annotated descriptors. As clinical information is present, practices with regard to de-identification are strictly adhered to. Documentations such as case report forms or study protocols pertaining to clinical trials are annexed accordingly. Advance users could extract data from the portal through application programming interfaces (APIs) while immunologists equipped with basic computational skills could query the database through a graphical user interface (GUI) via ImmPort Galaxy. The authors have performed a proof of concept usage of previous clinical trial data, by identifying distinct granulocytes subsets as predictors for treatment response to rituximab in patients with anti-neutrophil cytoplasmic antibody (ANCA) associated vasculitis (AAV) (138).

Recently, the same authors demonstrated how the ImmPort data can be utilized in a massive framework through the implementation of the 10,000 Immunomes Project (10KIP) (139). The project filtered and drew 10,344 healthy individuals from the original ImmPort database, of which more than 1,000 are pediatric subjects below the age of 18. The 10KIP project serves to provide a healthy immunological control reference dataset ranging across 10 types of information including, CyToF, flow cytometry, multiplex ELISA, gene expression, and clinical variates. The healthy dataset in 10KIP was compiled through manual curation of the original ImmPort dataset through a defined list of inclusion/exclusion criteria, consisting of samples prior to experimental manipulations (e.g., stimulations). Data is provided as either (a) “formatted” which consists of data that are harmonized for their analyte nomenclature and units of measurement, or (b) “normalized” which is additionally computationally corrected for batch variations to facilitate cross study comparisons. The authors illustrated the utility and robustness of the dataset and batch corrections by reaffirming previously proven age, gender or ethnic related immunological parameter (e.g., serum cytokines, cellular subsets) perturbations shown by other groups.

The ImmPort and the spin-off 10KIP provide an exemplary demonstration how the challenges associated with integrating diverse immunomics data can be surmounted. Despite these efforts, the authors have cited several limitations that persisted (139). Firstly, the compilation of independent datasets from the original laboratories is inadvertently dependent on the accuracy of the annotations (data descriptors or labeling). The veracity of the descriptors is entirely contingent on users who upload the datasets. Next, a key issue is with regard to the heterogeneous nature of how different laboratories may collect and analyze samples and this will likely contribute to the variance in the analytes measured. Despite the demonstration of the utility of batch correction normalization, it is performed based on assumptions that may be invalid for certain studies, which likely require further refinement. Lastly, datasets that are high in value but low in representation (e.g., numbers of RNAseq datasets were not sufficient) were omitted from inclusion in the 10KIP, a limitation dependent on user contribution or how well the platform has penetrated the community. The gradual implementation and uptake of such public databases which focus on viewing immunomics data as a whole will ultimately spur and allow tandem shifts in biological insights.

## Conclusion

The era of immunomics provides unprecedented access to platforms that encompass a wide array of capabilities to interrogate the complexity of pediatric rheumatic diseases. We have shown how various groups have tapped on these technologies to peer into the elaborate networks of immune cell subsets and related pathways, which have in turn given us important clues to pathological mechanisms. The resulting explosion of biological data has further presented the challenge of how to best integrate and assimilate such large amounts of data into a coherent narrative.

Nevertheless, the need to improve stratification and personalization of existing therapeutic regimens as well as to provide new treatments necessitate continued in-depth research into the immune profile of pediatric rheumatic diseases. This would demand appreciation of each immunomics platform's strengths and limitations to design complementary approaches for addressing important questions. Starting from a biological sample (e.g., blood, synovial fluid), deep immune phenotyping can be first performed in an unsupervised manner (e.g., mass cytometry), so as to obtain immune signatures of diverse cell subsets. To streamline high dimensional biological information regarding the sampled cells, computational algorithms can be put in place for dimensionality reduction and functional annotation to derive relevant immune signatures. Subsequently, populations of interest can be sorted for targeted downstream analyses (e.g., RNAseq, pathway analyses, epigenetics) reiteratively and accumulated data may then be functionally validated against clinical correlates. This proposed framework permits initial unbiased interrogation of the biological sample at the single cell level that is not possible with conventional technologies, and target cell subsets can then be evaluated individually or in bulk at different levels of gene expression (e.g., genomics, epigenomics, transcriptomics, cytomics). All in all, judicious use of immunomics platforms will unequivocally identify unique cellular signatures which compose the key to unraveling the mysteries of autoimmune disease.

In addition, it is imperative to maintain close interaction among researchers, clinicians, bioinformaticians, and technologists alike for continued evolution within the immunomics field, which will definitely provide exciting opportunities for all.

## Data Availability

No datasets were generated or analyzed for this study.

## Author Contributions

ST, KY, JL, JY, and TA contributed to the writing and conceptualization of the article. JL, TA, and SA helped in the revision of the manuscript.

### Conflict of Interest Statement

The authors declare that the research was conducted in the absence of any commercial or financial relationships that could be construed as a potential conflict of interest.
